# Preliminary evaluation of a brain PET insertable to MRI

**DOI:** 10.1186/2197-7364-1-S1-A13

**Published:** 2014-07-29

**Authors:** Gyuseng Cho, Yong Choi, Jae Sung Lee, Hyun Joon An, Jin Ho Jung, Hyun Wook Park, Chang Hyun Oh, Kyeongjin Park, Kyung Taek Lim, Minsik Cho, Woo Suk Sul, Hyoungtaek Kim, Hyunduk Kim

**Affiliations:** Department of Nuclear and Quantum Engineering, Korea Advanced Institute of Science and Technology, Daejeon, 305-701 South Korea; Department of Electronic Engineering, Sogang University, Seoul, 121-742 South Korea; Department of Nuclear Medicine, Seoul National University, Seoul, 110-744 South Korea; National NanoFab Center, Deajeon, 305-806 South Korea

There is a new trend of the medical image that diagnoses a brain disease as like Alzheimer dementia. The first qualified candidate is a PET-MRI fusion modality because MRI is a more powerful anatomic diagnosis tool than other modalities. In our study, in order to solve the high magnetic field from MRI, the development was consisted with four main items such as photo-sensor, PET scanner, MRI head-coil and attenuation correction algorithm development.

In the case of a silicon based Geiger-mode Avalanche Photo Diode (GADP), its pixel dimension is in 3 x 3 mm^2^. Fabricated GAPD has a high geometric fill-factor with quenching resistors of the high resistive poly-silicon layer and a high gain 10^6^. PET scanner was consisted of 72 detector modules arranged in a ring of 390 mm diameter. Each detector module was composed of a 4 x 4 array GAPD coupled with an array LYSO. The signals from each PET module were fed into preamplifiers using a 3 m long flat cable and outputs were fed into field programmable gate array (FPGA)-embedded data acquisition (DAQ) boards. A high-pass quadrature birdcage coil for high uniformity was developed in order to minimize the signal loss when it combines with PET module. In order to quickly and effectively fuse the taken image from each modality, we study a reconstruction and attenuation correction algorithm for PET images using MRI data. Finally, each component was integrated at the inside of 3 T MRI.

The preliminary test was performed while the PET-MRI system is operated simultaneously. We obtained a good performance of PET scanner that is the 16 % energy resolution and the 3.0 mm spatial resolution. Also the PET’s sensitivity in the center of field of view is a 1.2 %cps/Bq. In the case of a primary characteristic of MRI, the spatial resolution and the uniformity is a 1.0 mm (T1, T2) and 91 %(T1) / 87 %(T2) respectively.

The preliminary results indicate that the GAPD silicon photo sensor is excellently operated under the strong magnetic field and the developed PET-MRI system can provide high-quality PET and MRI images. Finally, an experiment of performance evaluation of a human brain of 3 candidates was conducted.

